# Type I IFN signaling shapes subset-specific monocyte fates in the injured myocardium

**DOI:** 10.1172/JCI209791

**Published:** 2026-09-01

**Authors:** Ecem Tugba Sakalli, Giuseppe Rizzo, Alma Zernecke, Gustavo Campos Ramos

**Affiliations:** 1 Institute of Experimental Biomedicine, University Hospital Würzburg, Würzburg, Germany.; 2 Department of Internal Medicine I / Comprehensive Heart Failure Centre, University Hospital Würzburg, Würzburg, Germany.

## Abstract

Monocytes and macrophages promote tissue repair following myocardial infarction, but the mechanisms tuning their effector functions remain elusive. While macrophages are essential in clearing debris and resolving inflammation, they can also contribute to uncontrolled inflammation and provoke additional damage. Thus, factors that influence macrophage differentiation trajectories and phenotypes play an important role in cardiac repair outcomes. By combining genetic lineage tracing with cell-specific targeting, the study from Koenig et al. sheds light on key signaling events that shape monocyte fate decisions in the injured myocardium, establishing a differentiation hierarchy among monocyte-macrophage subsets. The findings also reveal that macrophages with an IFN response signature give rise to MHCII^hi^ macrophages, which, in turn, contribute to regulatory T cell generation and cardioprotection. This work underscores the importance of understanding cardiac macrophage phenotypic plasticity within a broader framework of lineage relationships.

## Introduction

Macrophages, though initially defined by their phagocytic capacity (1), can display broad phenotypic and functional plasticity and contribute to both homeostatic and pathological processes (2). In the healthy myocardium, macrophages are the most abundant immune cell type and contribute to important housekeeping functions, including facilitating electrical conduction (3). Following myocardial infarction (MI), the local pool of tissue-resident macrophages is further complemented by infiltrating monocytes, which give rise to a broad range of cardiac macrophage subsets that shape tissue responses to acute injury (2). On the one hand, macrophages are important for clearing necrotic cell debris and for mediating acute inflammation, an indispensable step toward tissue repair. Yet, if uncontrolled, inflammatory responses can cause collateral damage and further cardiomyocyte death (4). This trade-off can be particularly costly for the adult mammalian heart, an organ with negligible regenerative capacity and low tolerance for disease (5). This Janus-faced cardio-immune crosstalk underscores the need for a deeper understanding of how macrophage phenotypic plasticity is regulated in the injured myocardium.

Studies over the past decade have shown that specialized subsets of cardiac macrophages can be distinguished into embryonic and adult hematopoietic lineages that are broadly defined by the surface expression of C-C chemokine receptor type 2 (CCR2), which is required for monocyte recruitment into inflamed tissues (6, 7). Cardiac-resident macrophages lacking CCR2 expression (and characterized by expression of CX3CR1) are enriched for cells of embryonic origin that play crucial roles in maintaining tissue homeostasis (2) and orchestrating tissue repair after MI (8). In contrast, CCR2^+^ monocytes are typically recruited to the injured myocardium and their progeny contribute to in situ inflammation (7). Several transcriptionally distinct monocyte and macrophage states have been identified in the healthy and infarcted heart (9–11), but the differentiation trajectories and relationships among different cardiac macrophage lineages remain poorly understood.

## Making sense of the cardiac macrophage diversity

**Figure 1:**
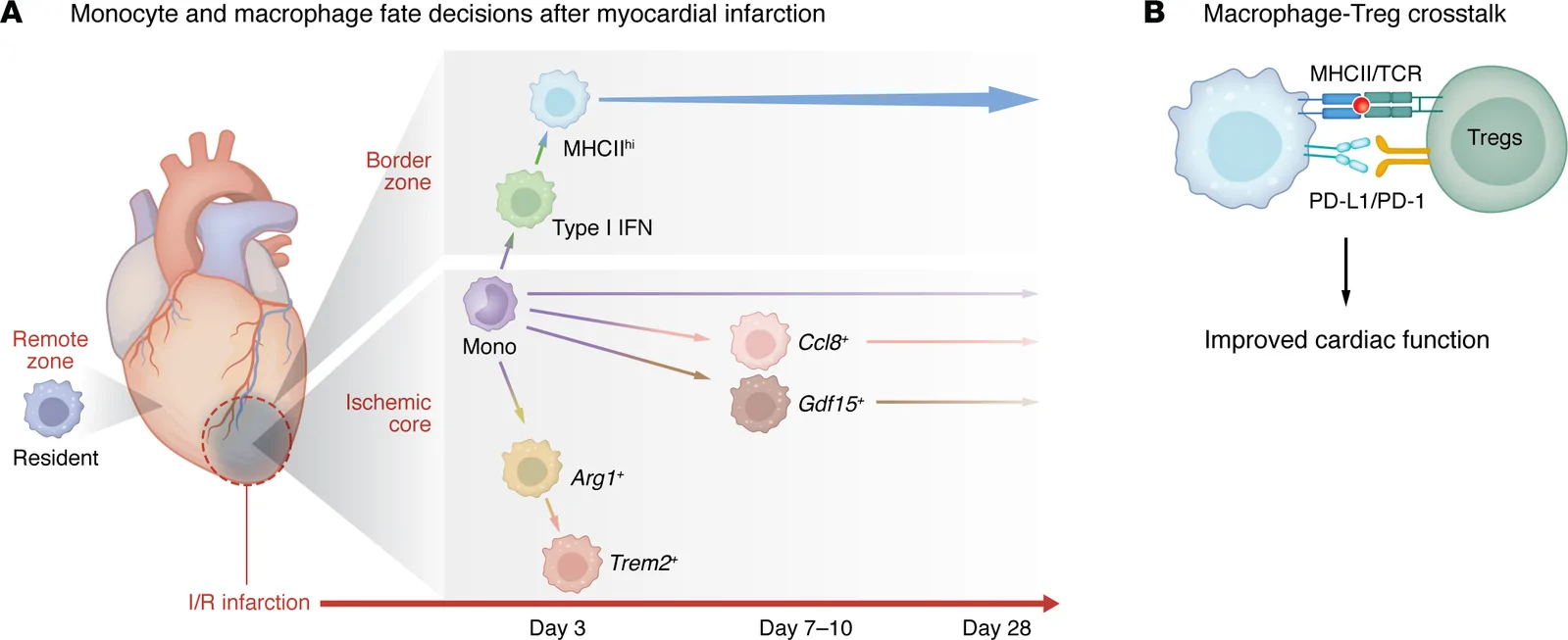
Monocyte-derived type I IFN-activated macrophages localize near the infarct border zone, giving rise to MHCII^hi^ macrophages that promote Treg development during cardiac remodeling after MI. (**A**) Koenig et al. (12) showed that monocytes differentiated into several macrophage subpopulations within days of injury, with a hierarchical relationship among subsets. Type I IFN–activated macrophages represent an intermediate state that localized near the infarct border zone and terminally differentiated into MHCII^+^ macrophages that persisted in the heart through 28 days after injury. (**B**) Additionally, MHCII^+^ macrophages contributed to cardiac repair after MI by promoting Treg development in the ischemic heart.

In a recent study, Koenig et al. shed light on key events shaping macrophage differentiation in the infarcted myocardium by combining murine genetic models for lineage tracing and cell-specific ablation tools. The authors used *Ccr2*^creERT2^Rosa26LSL-^tdTomato^ mice to specifically label monocytes and monocyte-derived macrophages, combined with single-cell RNA sequencing (scRNA-seq) of genetically labeled cells that were purified from the heart at different time points after MI. This approach enabled the authors to dissect the temporal dynamics of recruited monocytes, establish their differentiation trajectories, and uncover lineage relationships among their progeny (12). During the acute stage, monocyte-derived *Arg1*^+^ macrophages and type I IFN–activated macrophages were identified as intermediate states that give rise to *Trem2^+^* macrophages and MHCII^hi^ macrophages, respectively, which are conserved in humans and, consistent with previous literature, represent terminally differentiated states (9, 10). Furthermore, spatial transcriptomics showed that *Arg1^+^* and *Trem2^+^* macrophages were localized in the infarct region, whereas type I IFN–activated macrophages and MHCII^hi^ macrophages were primarily found at the infarct border zone (12). Using a pulse-chase strategy in *Cx3cr1*^CreERT2^*Rosa26*^LSL-tdTomato^ mice and specific labeling of *Cx3cr1*^+^ resident macrophages, Koenig et al. could further confirm that resident macrophages did not contribute to these macrophage subsets (12). Previously, a monocyte and monocyte-derived macrophage depletion approach using an anti-CCR2 antibody showed that the diverse macrophage subsets in the infarcted heart were derived from recruited monocytes rather than from homeostatic tissue-resident macrophages (10). These observations provide an innovative framework for establishing lineage relationships among the various cardiac macrophage subsets and delineating the hierarchy of transient and terminally differentiated states (Figure 1A).

A particularly interesting finding in the study by Koenig et al. emerged from their examination of the early fate decisions underlying monocyte-macrophage differentiation already during tissue extravasation (12). Intravenous anti-CD45 antibody labeling 5 minutes prior to termination to label intravascular leukocytes, combined with flushing of cells that have not adhered to the endothelium at harvest, further revealed that monocyte-to-macrophage differentiation had already begun during monocyte extravasation into the infarcted tissue (12). This observation introduces the concept that macrophage cell-state specification does not occur solely within the infarcted heart but begins during extravasation. For future research, it would be highly interesting to further investigate how extravasation affects specific monocyte differentiation trajectories (13, 14) and, in particular, how the biological niche and interactions between immune and stromal cells, such as endothelial cells and pericytes, regulate monocyte-to-macrophage fate determination.

## Cell-specific effects of type I IFN signaling in the infarcted myocardium

Although the importance of the IFN response in immunity has been well established in the context of antiviral defense and autoimmunity (15), its complex roles in cardiovascular diseases remain to be fully elucidated (14, 16–18). In both human and experimental MI models, ischemic injury has been shown to trigger an IFN response in monocyte and neutrophil progenitors already in the bone marrow (14). Moreover, the systemic effects of a type I IFN response in the context of MI were investigated, demonstrating that global suppression of IFN regulatory factor 3 (*Irf3*) improved cardiac remodeling and survival after MI in mice (16). To further examine the cell-specific roles of the type I IFN response, the effects of selective *Irf3* deletion across different cell types, including cardiomyocytes, fibroblasts, macrophages, neutrophils, and endothelial cells, were studied, and indicated that only cardiomyocyte-specific *Irf3* deficiency reduced IFN-stimulated gene expression and colonies of IFN-induced cells, while deletion in leukocytes did not exert any effects (17).

Koenig et al. further investigated the cell-specific effect of the IFN response by selectively deleting *Ifnar1*, encoding a subunit of the cell-surface receptor for type I interferons in recruited monocytes. This deletion led to a significant reduction in the MHCII^hi^ macrophage population, indicating that type I IFN–activated macrophages and type I IFN signaling are the major contributors to this population (12). Unexpectedly, the authors observed a worsened cardiac adverse remodeling in mice with monocyte and monocyte-derived macrophage specific *Ifnar1* ablation, demonstrating that type I IFN signaling in infiltrating monocytes and their progeny confers a protective effect on MI remodeling (12). Mechanistically, this phenotype was associated with enhanced infiltration of Tregs into the myocardium, (12) with MHCII expression by monocyte-derived macrophages mediating this protective response following MI. Furthermore, among cardiac macrophages, type I IFN–activated macrophages expressed PD-L1 (programmed death-ligand 1), an immune checkpoint ligand that promotes immune tolerance by engaging PD-1 on T cells, which may have contributed to Treg differentiation. By linking Type I IFN macrophages to Treg priming, these observations provide what we believe to be the first evidence that type I IFN–activated macrophages can foster Treg expansion associated with cardioprotective effects. Previous studies have shown that myocardial Treg cells can suppress myocardial inflammation and contribute to myocardial protection (19). Along these lines, Treg ablation leads to increased proinflammatory myeloid cell recruitment and overt myocardial inflammation (20, 21). The study by Koenig et al. expands this picture by highlighting that Treg-macrophage crosstalk is bidirectional (Figure 1B), opening interesting avenues for immunoregulation.

## Conclusion and future directions

Because inflammatory mechanisms can exert both beneficial and detrimental effects on healing after MI, future approaches are required to target inflammatory mechanisms linked to collateral damage without disturbing immune cells’ critical roles in promoting tissue repair. The study by Koenig et al. contributes to an in-depth understanding of the cardiac macrophage dynamics by describing distinct transcriptional and spatially resolved states over the course of repair after MI. In addition, they report that key events defining monocyte fate decisions already begin during their extravasation and infiltration into the infarcted tissue. Importantly, these findings reveal the complex nature of the type I IFN response and its diverging cell-specific effects, which, in specific circumstances, can also contribute to cardiac repair by fostering Treg differentiation.

## Conflict of interest

GCR is coinventor on a pending patent (EP26192875.8) application with focus on engineered T cells. The remaining authors have declared that no conflict of interest exists.

## Funding support

- The German Research Foundation (Collaborative Research Centre 1525 ‘Cardio-Immune Interfaces’, grant #453989101, projects A3/ B6 to GCR, A1/ C6 to AZ, and PS2 to GR).
- The Heisenberg Program, German Research Foundation (grant #517001338 to GCR).

## Version 1 09/01/2026

Electronic publication
